# Low Intensity Focused Ultrasound (LOFU)-mediated Acoustic Immune Priming and Ablative Radiation Therapy for *in situ* Tumor Vaccines

**DOI:** 10.1038/s41598-019-51332-4

**Published:** 2019-10-29

**Authors:** Karin A. Skalina, Saurabh Singh, Claudia Gutierrez Chavez, Fernando Macian, Chandan Guha

**Affiliations:** 1 Departments of Pathology, Albert Einstein College of Medicine, Bronx, NY USA; 2 Urology, Albert Einstein College of Medicine & Montefiore Medical Center, Bronx, NY USA; 3 Radiation Oncology, Albert Einstein College of Medicine & Montefiore Medical Center, Bronx, NY USA; 4 Institute for Onco-Physics, Albert Einstein College of Medicine, Bronx, NY USA

**Keywords:** Cancer immunotherapy, Immunosurveillance, Radiotherapy, Tumour immunology, Prostate cancer

## Abstract

Focal ablative therapies have been primarily used for local tumor ablation. However, they often fail to impact systemic disease. Here we propose the use of low intensity focused ultrasound (LOFU), a noninvasive, nontoxic, conformal therapy, to deliver acoustic stress to the tumor for immune priming. We demonstrate that LOFU significantly induces expression and cell surface localization of heat shock proteins in murine breast (4T1) and prostate adenocarcinoma (TPSA23) cancer cell lines. *In vivo* LOFU followed by ablative radiation therapy (RT) results in primary tumor cure, upregulation of a cytotoxic immune response and induction of immunological memory by inhibiting secondary tumor growth upon re-challenge with tumor cells. We, therefore, describe a regimen of a combination therapy with noninvasive, acoustic immune priming and ablative radiation therapy to generate an *in situ* tumor vaccine, induce CD8+ T cells against tumor-associated antigens and provide a viable oncologic treatment option for solid tumors.

## Introduction

Focal ablative therapies using physical energy, such as ionizing radiation, photodynamic therapy, hyperthermia, high-intensity focused ultrasound (HIFU) and radiofrequency ablation, can produce local control of tumors but usually do not affect systemic cure. Recently, several groups have shown that treatment with several forms of physical energy can cause immunogenic cell death (ICD) of tumors, thereby making them attractive partners for combination with immunotherapeutics^1^. Kroemer and colleagues initially discovered that several chemotherapeutic agents and ionizing radiation induce ICD in tumor cells, which is characterized by the induction of endoplasmic reticulum (ER) stress response, generation of reactive oxygen species (ROS), activation of danger-associated molecular pattern (DAMP) signals and induction of protective anti-tumoral immunity which prevents tumor growth^2,3^. In contrast, tumor ablation without ICD failed to generate a protective systemic immune response, thereby suggesting that the release of tumor antigens from dying cells without appropriate DAMP signals is immunologically silent. Even ICD-inducing ablative procedures, such as radiation therapy (RT), usually fail to generate systemic anti-tumoral immunity and eradicate metastatic disease. A critical factor leading to the lack of systemic cure from focal tumor ablative therapies is inefficient antigen presentation to CD8+ T cells in the tumor microenvironment. Processing of extracellular tumor-derived antigens into peptides and cross-presentation of neo-epitopes onto cell surface class I MHC molecules by antigen presenting cells (APC), such as dendritic cells (DCs), is critical for activation of cytotoxic CD8+ T cells. In order to consistently induce anti-tumoral immunity with focal tumor ablation, such as, RT, we have devised a strategy of “immune priming” with non-ablative, low-energy or low-intensity focused ultrasound (LOFU), followed by tumor ablation by RT to generate an *in situ* tumor vaccine that induces anti-tumoral immunity.

HIFU has been recently approved by the Food & Drug Administration (FDA) for the ablation of prostate tissue, including localized prostate cancer, which is the second leading cause of cancer-related deaths in the United States^4,5^. Currently, however, there are minimal effective therapies for metastatic prostate cancer, which has a 28% 5-year survival rate^6^. Most patients who receive HIFU treatment of solid malignancies have either local recurrence^7^ or systemic metastases that develop after treatment^8^. HIFU causes instantaneous necrotic cell death at the focal point and the release of denatured proteins from these cells might not be efficient at generating a robust anti-tumoral T helper 1 (Th1) and cytotoxic T cell (CTL) mediated immune response. The peripheral zone of HIFU-ablated tissue, which receives heat diffusion from the ablated zone, exhibits increased expression of heat shock proteins (HSP) and infiltration of immune effector cells, including CD8+ CTLs and CD11c+ APCs^9,10^. HSPs are highly conserved chaperone proteins that bind to the hydrophobic domains of peptides and misfolded proteins. DCs engulf extracellular HSP-peptide complexes released from dying tumor cells and cross-present these peptides on cell surface class I MHC molecules to activate CD8+ T cells^11,12^. We have devised a LOFU treatment that produces mechanical and thermal stresses in cells transiently without killing them. LOFU is different from hyperthermia in that the ultrasound pulse is delivered over a short period of time of 1.5 seconds per focal spot, instead of the 30–90 minutes for hyperthermia. We reasoned that the acoustic stress generated by LOFU should produce protein misfolding, ER stress and thus stimulate the expression of HSP genes. Therefore, we hypothesized that LOFU-mediated immune priming of tumors, followed by ablative RT should increase the release of tumor-derived HSP-peptide complexes that could promote antigen cross-presentation and activation of CD8+ T cells for the induction of systemic anti-tumoral immunity. We previously demonstrated that LOFU could reverse tumor-induced T cell anergy in tumor draining lymph nodes and enhanced local, regional and systemic control of metastatic melanoma^13^. In this report, we demonstrate that LOFU induces a heat shock protein response in murine breast and prostate cancer cell lines and the combination therapy of LOFU and ablative RT controls primary murine prostate cancer, while increasing anti-tumoral cytotoxic T cell response and immune memory in a murine prostate cancer model.

## Results

### LOFU increases the expression and cell surface localization of heat shock proteins (HSP)

**Figure 1:**
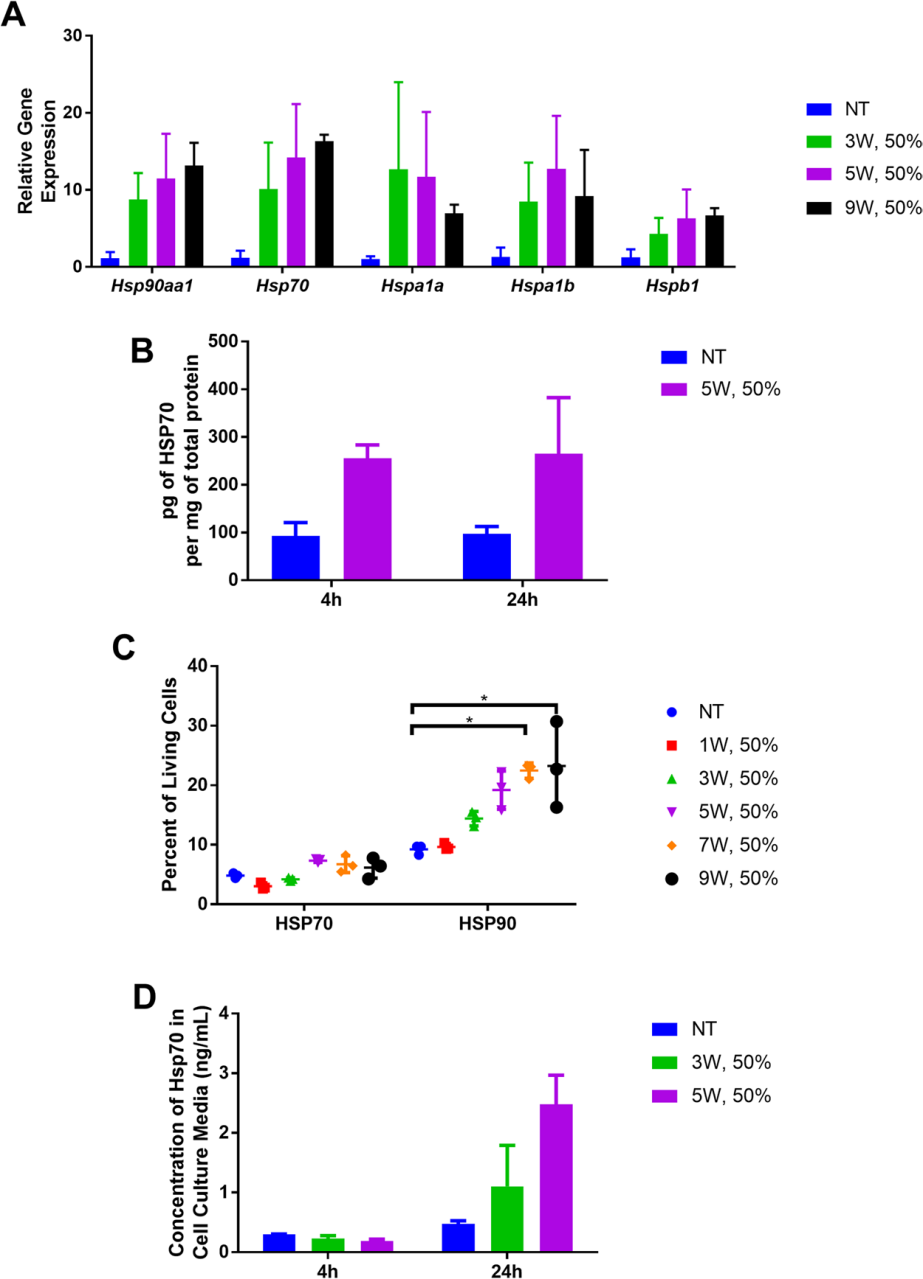
LOFU modulates the expression and cellular distribution of *Hsp* gene family members in 4T1 breast cancer cells. (**A**) LOFU augments the expression of *Hsp* RNA. qRT-PCR was performed on cell lysates, isolated 4 hours after treatment of 4T1 cells, (ANOVA *Hsp70* p = 0.05, *Hspb1* p = 0.05). Figure is representative of 3 independent experiments. (**B**) LOFU increases cellular HSP70 protein concentration. HSP70 ELISA was performed with total cell lysate, obtained 4 hours and 24 hours after LOFU treatment. (**C**) LOFU induces translocation of cytosolic HSP proteins to the cell surface. Flow cytometric analysis of cell surface expression of HSP70 and HSP90 was performed 4 hours after LOFU treatment (ANOVA HSP70 p = 0.02, HSP90 p = 0.01). (**D**) LOFU increase HSP70 protein secretion. HSP70 protein concentration in the cell culture medium, obtained 4 hours and 24 hours after LOFU treatment was measured by ELISA.

We analyzed the expression of HSP mRNA and protein localization in LOFU-treated, mouse breast and prostate cancer cell lines, 4T1 and TPSA23, respectively. We first determined the effects of varying low intensities (I_SATP_ < 800 W/cm^2^) of ultrasound on Hsp gene expression in 4T1 cells, a mouse model of triple negative breast cancer. Quantitative RT-PCR (qRT-PCR) analysis using primers for Hsp gene families showed that there were significant increases in mRNA levels across all family members with Hsp70 and Hsp90aa1 RNA displaying the highest expression (13–16 fold over non-treated), when normalized to Gapdh RNA expression, with increasing intensity of LOFU, four hours after treatment (Fig. 1A). To examine whether LOFU treatment increased cytoplasmic HSP70 protein levels, we performed HSP70 ELISA of cell lysates. There was a significant increase from 93.13 ± 27.8 to 255.3 ± 28 pg of cytosolic HSP70 per mg of total protein, four hours after LOFU treatment (Fig. 1B). Since the cell membrane is the first to encounter ultrasound pulses, we therefore examined cell surface localization of HSP70 and HSP90 on 4T1 by flow cytometry as a measure of acoustic stress. The translocation of cytoplasmic HSPs to the cell surface also provides an activation signal for natural killer cells and danger signals for DC activation^14,15^. Cell surface HSP70 increased after treatment with 5 W, 50% duty cycle (7.3% of cells having surface HSP70 compared to 4.8% in non-treated). For HSP90, the surface localization also peaked with 5W, 50% duty cycle (19.2% versus 9.3% non-treated) before reaching a plateau with higher intensity treatments (22.5% and 23.2% with 7W, 50% and 9W, 50% respectively) (Fig. 1C). Lastly, we measured the secretion of HSP70 in the culture supernatant of 4T1 cells by ELISA 4 hours and 24 hours after LOFU treatment. Four hours after LOFU, there was no evidence of HSP70 or HSP90 secretion. However, 24 hours after treatment, there was an increase in HSP70 secretion by LOFU-treated cells, compared to untreated cells (2.5 ng/mL versus 0.476 ng/mL, respectively) (Fig. 1D).

**Figure 2:**
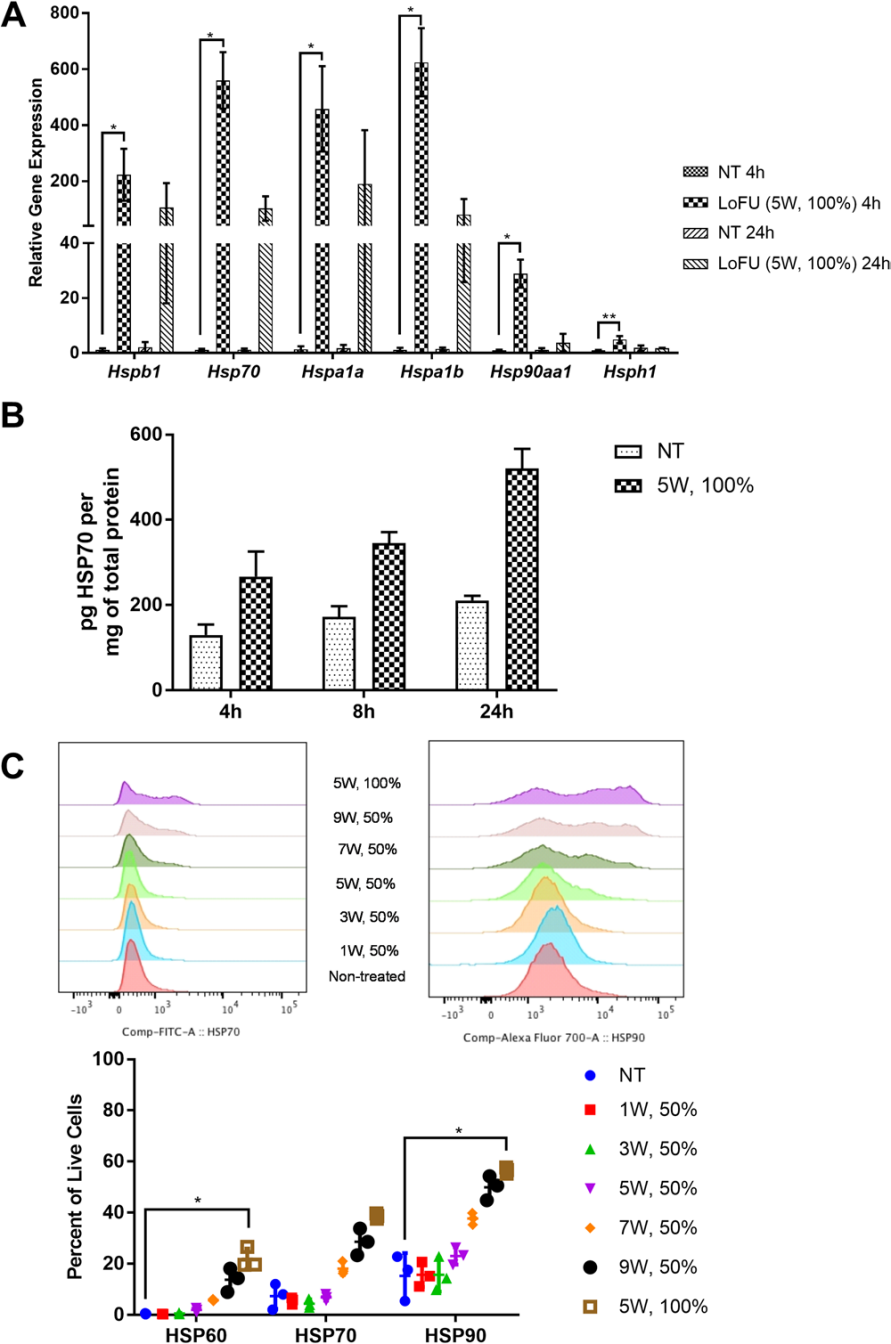
LOFU modulates the expression and cellular distribution of *Hsp* gene family members in TPSA23 prostate cancer cells. (**A**) LOFU augments the expression of *Hsp* RNA. qRT-PCR was performed on cell lysates isolated 4 hours and 24 hours after LOFU treatment (5 W, 100% duty factor) of TPSA23 cells (ANOVA *Hspb1* p = 0.003, *Hsp70* p = 0.001 *Hspa1a* p = 0.003, *Hspa1b* p = 0.001, *Hsp90aa1* p = 0.007, *Hsph1* p = 0.001.) Figure is representative of 3 independent experiments. (**B**) LOFU increases cellular HSP protein synthesis. HSP70 ELISA was performed with cell lysates 4 hours, 8 hours and 24 hours after treatment with LOFU (5 W, 100% duty factor). (**C**) LOFU induces translocation of cytosolic HSP proteins to the cell surface with increasing intensity. Flow cytometry analysis of surface expression of HSP60, HSP70 and HSP90 was performed 4 hours after LOFU treatment. (ANOVA HSP60 p = 0.005, HSP70 p = 0.009 HSP90 p = 0.008).

TPSA23 is a TRAMPC1-derived tumor cell line, which expresses human prostate specific antigen (PSA)^16^. In TPSA23 cells, Hsp RNA expression was increased across all families of Hsp genes (Fig. 2A) within 4 hours of LOFU. Hspa1b (HSP70) mRNA showed the greatest increase (624.5 ± 121.3-folds) over non-treated group (p = 0.02). Hspa1a (HSP72) closely followed with a 458.3 ± 152-fold change (p = 0.03) in RNA expression. LOFU treatment of TPSA23 cells significantly increased the expression of mRNAs of Hspb1 (HSP27) by 222.8 ± 92.8-folds (p = 0.03), Hsp90aa1 (HSP90α) by 28.9 ± 5.1-folds (p = 0.04), and Hsph1 (HSP110) by 4.9 ± 1.3–folds (p = 0.007) over non-treated cells, four hours after LOFU treatment. The gene expression changes also translated into a 2.5-fold increase in HSP70 protein expression at 24 hours, as demonstrated by an ELISA of HSP70/HSPA1A (Fig. 2B). LOFU alone also increased the HSP70 concentration in lysates of *in vitro* treated TPSA23 cells, 4 hours, 8 hours and 24 hours after treatment (Fig. 2B). We, then analyzed the surface localization of HSP60, 70 and 90, four hours after LOFU treatment of cells, using flow cytometry, (Fig. 2C). Surface HSP90 increased the most after LOFU treatment (15.2% ± 8.9, untreated to 56.7% ± 1.2, LOFU-treated), followed by HSP70 (7.4% ± 5, untreated to 38.7% ± 1.1, LOFU-treated), and HSP60 (0.45% ± 0.19, untreated to 22.4% ± 4, LOFU-treated). Together these results indicate that a short pulse (1.5 sec) of LOFU induced a substantial acoustic stress response with modulation of HSP RNA expression and protein localization in 4T1 and TPSA23 tumor cells. The cell surface translocation of HSPs are more pronounced in TPSA23 cells, compared to 4T1 cells indicating that differences in the intrinsic biology of tumor types may determine the extent of cell surface translocation of HSPs. The cell surface HSPs post-LOFU treatment could provide danger signals for DC activation and induction of tumor-specific immune response^15^.

### LOFU immune priming of radiation therapy (RT) for TPSA23 tumors

**Figure 3:**
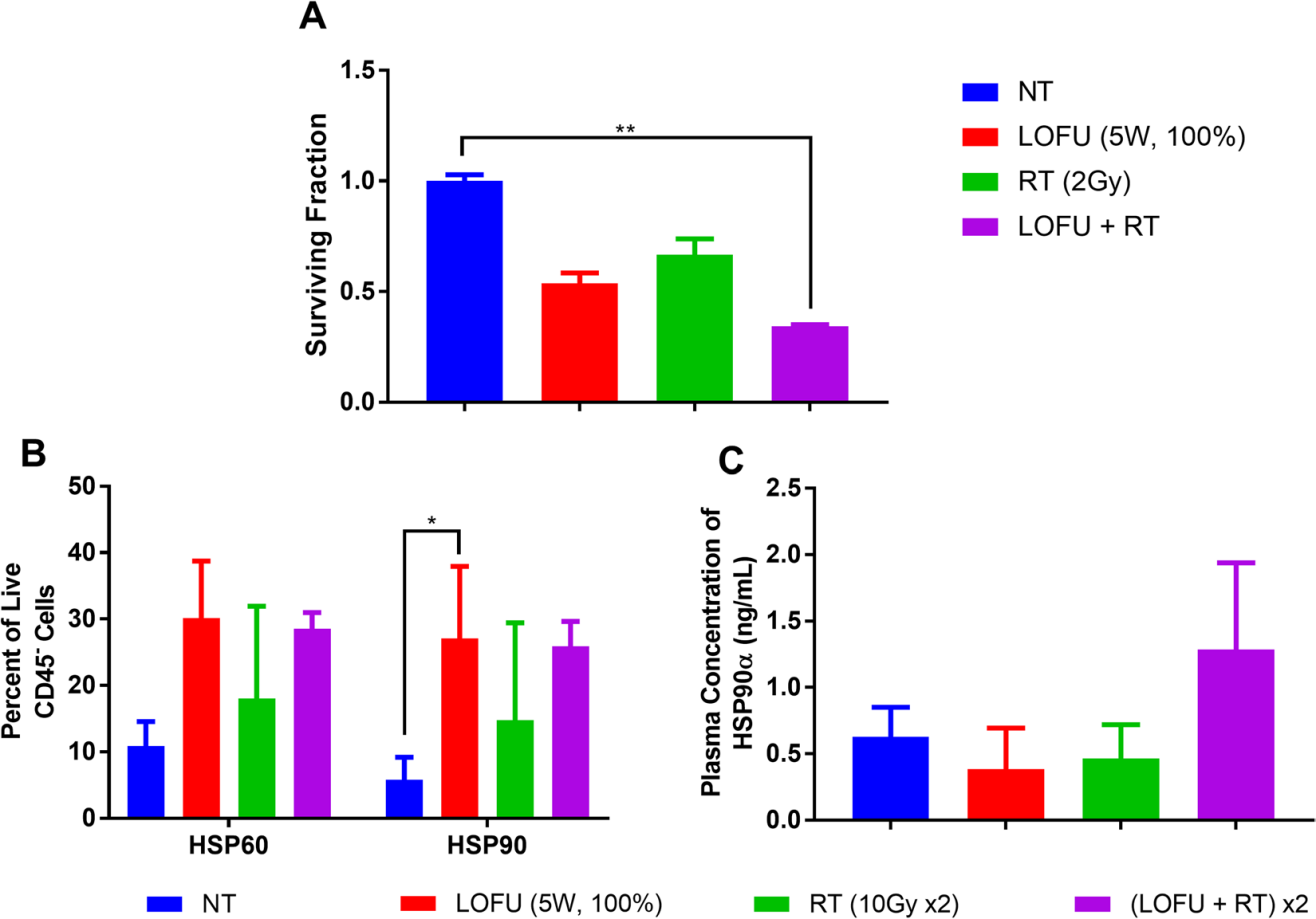
Combination LOFU + RT reduces clonogenicity and enhances HSP response *in vivo*. (**A**) LOFU + RT reduced clonogenic potential of TPSA23. Clonogenic assay following LOFU and radiation demonstrating decreased surviving fraction with combination treatment. Five hundred cells were plated immediately after LOFU treatment and radiation was performed after 2–3 hours of incubation. Colonies were fixed and stained with crystal violet after 7 days of incubation. (ANOVA p < 0.0001). (**B)** LOFU increases surface localization of HSPs *in vivo*. Percent of HSP60 and HSP90 on the surface of tumor cells 24 hours after treatment *in vivo* shows an increase in HSP60 and HSP90 post-LOFU treatment not by RT alone. (ANOVA HSP90 p = 0.04). (**C**) LOFU + RT induces HSP90α release into the plasma. Concentration of HSP90α in the plasma of mice 24 hours after treatment shows an increase in soluble HSP90α only post-LOFU plus RT indicative of increased immunogenic cell death.

As demonstrated in Figs 1 and 2, both 4T1 and TPSA23 cell lines showed increased expression of HSPs at the mRNA and protein levels after LOFU treatment. However, TPSA23 showed a more robust response, hence we chose TPSA23 as a tumor model for further *in vivo* studies. We selected a LOFU treatment of 1 MHz frequency, 100% duty factor, 5 W and 1.5 second treatment time per focal spot for optimal immunomodulation without increasing the cytotoxicity at higher LOFU intensities. Therapeutic ultrasound has also been shown to inhibit phosphorylation and activation of STAT3 in prostate cancer cells, transiently for 6 hours^17–19^. Since phopho-STAT3 can induce radio-resistance^20–23^, we chose to deliver RT fractions, 2–4 hours after LOFU treatment of TPSA23 tumors. Thus, we hypothesized that LOFU will transiently make TPSA23 cells radiosensitive and induce a heat shock response in TPSA23 tumors with an increase in the expression, cell surface localization and secretion of HSP proteins. Ablative RT after LOFU treatment would increase the release of tumor derived peptide-HSP complexes for cross-presentation by antigen presenting cells, thereby, increasing the immunogenicity of tumor cells and tumor growth retardation. In order to study the effect of combining LOFU treatment to ablative RT, we first determined whether LOFU inhibits the clonogenic capacity of irradiated TPS23 cells. Figure 3A shows the results of a clonogenic assay after LOFU alone, RT (2 Gy), and LOFU + RT. Combination therapy resulted in a reduction in the surviving fraction of cells, when compared to each individual treatment and radiation alone, indicating that LOFU enhances the tumoricidal effects of RT by multiple mechanisms, including inhibition of STAT3 activation.

To confirm our *in vitro* results of an increased cell surface HSPs after LOFU treatment, we further evaluated the cell surface expression of HSP60 and 90 by flow cytometry in LOFU-treated TPSA23 tumors *in vivo* (Fig. 3B). LOFU alone and LOFU + RT treatment showed a higher percentage of HSP60 and HSP90 expressing tumor cells, consistent with the *in vitro* results. Finally, we tested whether LOFU increases the secretion of HSPs from irradiated tumor cells. Figure 3C shows that only after combination treatment of LOFU + RT was there an increase in the plasma concentration of HSP90α (1.285 ± 0.652 pg/mL) when compared to monotherapy (LOFU alone: 0.386 ± 0.309 pg/mL; RT alone: 0.466 ± 0.254 pg/mL) or nontreated (0.628 ± 0.224 pg/mL) control groups. These results raise the possibility that acoustic immune priming by LOFU could increase the immunogenic potential of RT by providing HSP-chaperoned peptide antigens and danger signals released from dying irradiated tumor cells, to activate both the innate and adaptive immune response against tumors.

### LOFU + RT cures primary prostate tumor in a T cell dependent manner

**Figure 4:**
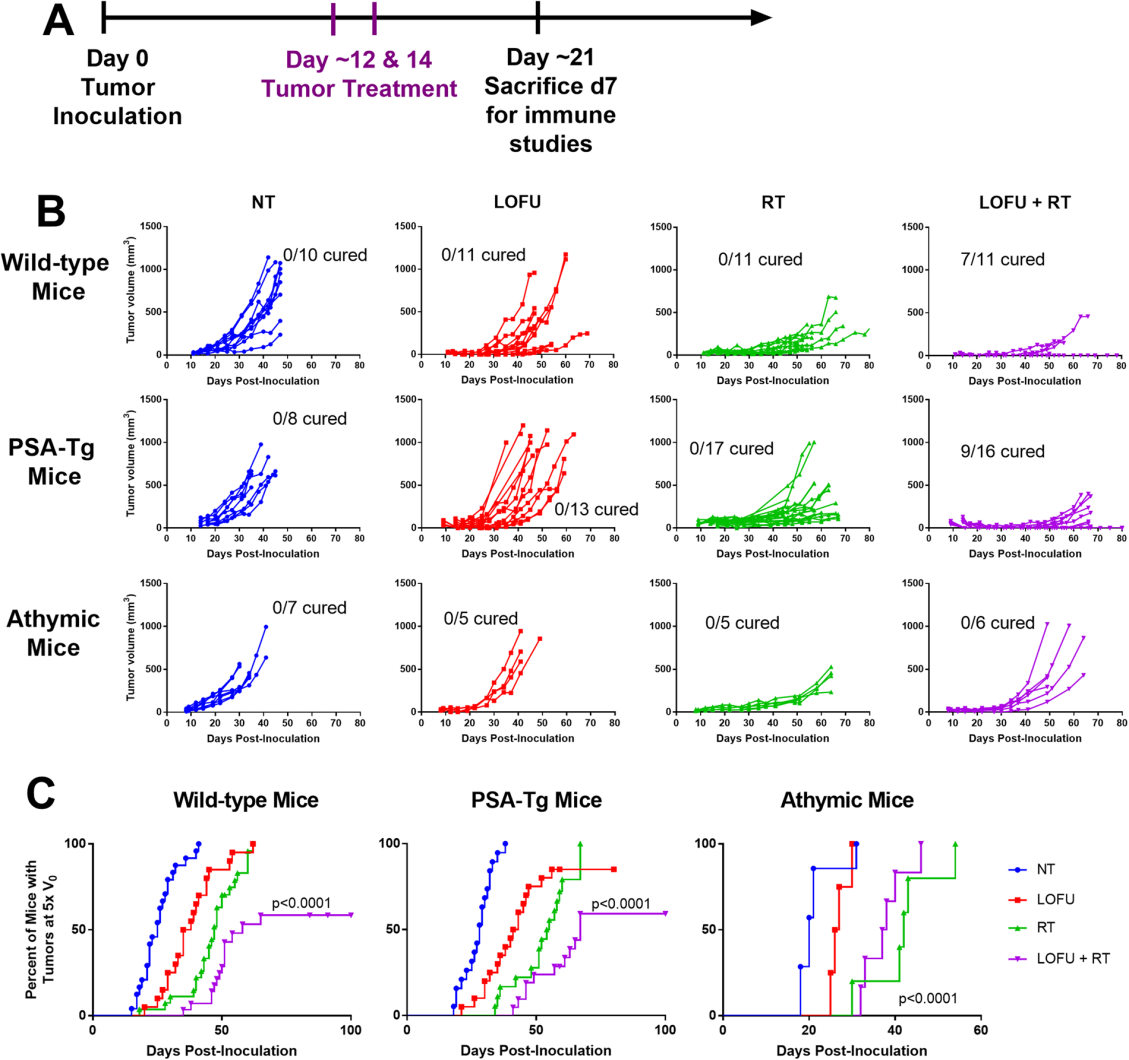
LOFU + RT significantly inhibits tumor growth in a T-cell dependent manner. (**A**) *In vivo* treatment scheme for TPSA23. LOFU treatment was performed about 12 days after tumor inoculation when the tumors were 4–6 mm in diameter. 10 Gy of radiation was performed about 2 hours after LOFU treatment. These treatments were repeated two days later, on day 14 post-inoculation. (**B**) LOFU + RT retards primary tumor growth in T cell dependent manner. Individual tumor volume curves of TPSA23 in wild-type male C57BL/6 mice (top row), 10–11 mice/group from two independent experiments; in male PSA-Tg mice (middle row), 8–17 mice/group from three independent experiments; in athymic nude male mice (bottom row), 5–7 mice/group. (**C**) 50% of tumors treated with LOFU + RT in immunocompetent mice do no reach 5 times the initial volume. time to reach 5 times the initial volume (V_0_), WT (left): no treatment (NT) n = 24, LOFU n = 20, RT n = 27 and LOFU + RT n = 28; PSA-Tg (center) NT n = 12, LOFU n = 14, RT n = 12, LOFU + RT n = 14; athymic nude (right): NT n = 7, LOFU n = 5, RT n = 6, LOFU + RT n = 6; significance determined by log-rank test.

In order to study whether LOFU-induced immune priming followed by ablative RT can induce anti-tumoral immunity and improve tumor control, we used a murine model of prostate cancer, TPSA23, grown either in wild-type male C57BL/6 mice, prostate specific antigen (PSA) transgenic mice^24^, or in immunodeficient athymic nude mice. We used PSA as a tumor antigen because PSA vaccines have been designed for immunotherapy for prostate cancer^25^. Since PSA is a foreign protein in C57BL/6 mice, we are also studying the effects of LOFU in PSA transgenic mice to determine whether LOFU can induce an immune response in animals tolerant to PSA as a self-antigen. Tumor growth in wild-type male C57BL/6 mice was significantly inhibited in the LOFU + RT combination therapy group, with 46% of mice (n = 28) never reaching 5 times the tumor volume at the start of treatment (V_0_) (Fig. 4B-top, C-left). Tumor volumes of wild-type mice in the RT alone and LOFU + RT combination group were significantly reduced compared to the non-treated group at day 47 post-inoculation, the latest time point at which all mice were still alive (p = 0.03, and 0.0007 respectively, Dunn’s multiple comparisons test). Additionally, with the combination of LOFU and RT resulted in a significant number of primary tumor cures (7 out of 11 mice), whereas RT alone did not cure any mice (Fisher’s exact test, p = 0.0039). These results suggest that ultrasound treatment prior to RT is required for successful local tumor control.

To determine if our LOFU + RT combination therapy could overcome tolerance to self-antigen, we used the TPSA23 cell line in transgenic mice expressing human PSA in the prostate^24^. Our results demonstrated that combination therapy of LOFU and radiation was able to overcome or bypass tolerance and successfully cure the primary tumor in over 50% of the mice treated (Fig. 4B-middle, C-center). When compared to RT alone, LOFU + RT achieved more complete response of tumors (0/17 cures in RT versus 9/16 cures in LOFU + RT groups, Fisher’s exact test p = 0.0003). On day 35 post tumor inoculation, only LOFU + RT and no LOFU or RT alone had tumor growth retardation compared to non-treated (p = 0.001 by Dunn’s multiple comparisons test). To determine whether the efficacy of LOFU + RT required T lymphocytes, identical experiments were conducted in athymic nude mice. The combination treatment of LOFU + RT demonstrated no enhancement in the tumor growth retardation over radiation alone (194.1 ± 106.7 mm^3^ and 104.4 ± 36.6 mm^3^, respectively, p = 0.13 Mann-Whitney U on day 41) indicating involvement of T lymphocyte mediated antitumor immune response elicited by LOFU + RT combination (Fig. 4B-bottom, C-right).

### LOFU + RT augments anti-tumoral cytotoxic T cell immunity

**Figure 5:**
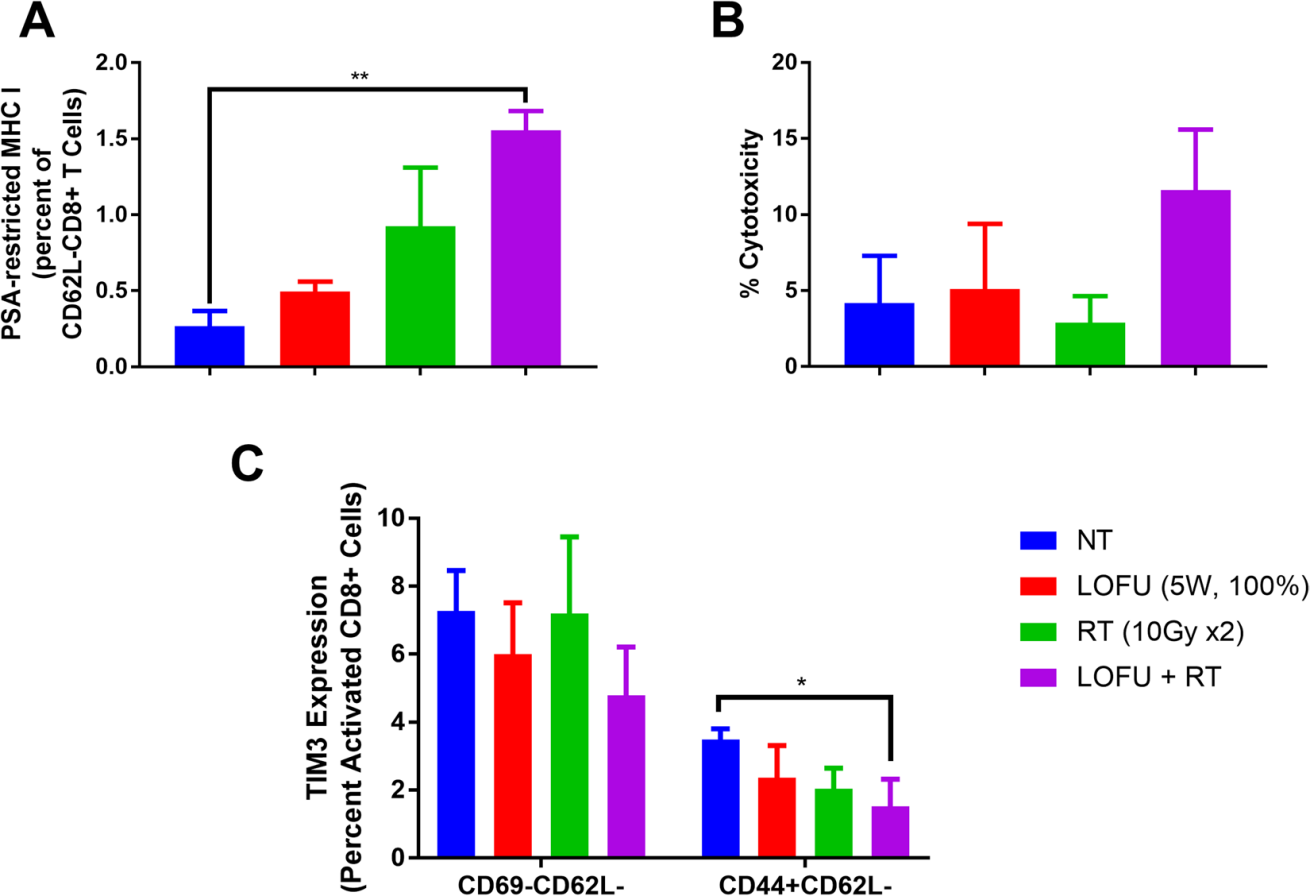
LOFU + RT increases anti-tumor immunity. (**A**) LOFU + RT induces PSA-specific activated CD8+ T cells. Splenic PSA-MHC class I restricted pentamer + activated (CD62L-) CD8+ T cells increased in wild-type mice 7 days after the end of treatment. (ANOVA p < 0.0001) (**B**) LOFU + RT enhances tumor-specific T cell mediated death. Increased T cell-mediated toxicity in splenocytes isolated 7 days after the end of treatment in PSA-Tg mice as measured by LDH cytotoxicity assay. (ANOVA p = 0.05) (**C**) LOFU + RT reduces splenic exhausted CD8+ T cells. Exhausted (TIM3+) activated (CD69-CD62L-) and effector memory (CD62L-CD44+) CD8+ T cells in spleen reduced 7 days after combination treatment. (ANOVA CD44 + CD62L- CD8+ T cells p = 0.05).

To determine the impact of combination therapy on systemic CD8+ T cell responses, we analyzed tumor antigen specific T cells in splenocytes 7 days after the completion of primary tumor treatments, using a PSA-specific MHC Class I pentamer. There was an increase in the PSA-specific, activated (CD62L-) CD8+ T cells across all the treatment groups in WT mice, with LOFU + RT having the highest percentage of CD62L-/pentamer + CD8 T cells (1.557 ± 0.127%) compared to LOFU alone (0.497 ± 0.064%) and RT alone (0.923 ± 0.387%) groups (Fig. 5A). Similar experiments were performed in PSA-Tg mice, which did not show a significant increase in PSA-specific CD8+ T cells with LOFU + RT (data not shown). These results indicate that the tolerance to PSA was most likely circumvented as indicated by the LDH release to tumor lysate. An LDH release assay indicative of immune-mediate cytotoxicity also confirmed that splenocytes from PSA-transgenic mice treated with the combination therapy were able to kill more naïve tumor cells (NT: 4.186 ± 3.1%, LOFU: 5.1 ± 4.3%, RT: 2.91 ± 1.7%, LOFU + RT: 11.613 ± 3.98%; Fig. 5B). Additionally, reduction of TIM3 expression on the surface of activated (CD62L-CD69-) and effector memory (CD44 + CD62L-) CD8+ T cells was significantly reduced in spleens of combination treated PSA-Tg mice 7 days after the end of treatment (Fig. 5C).

### LOFU + RT enhanced immune memory in a tumor rechallenge model

**Figure 6:**
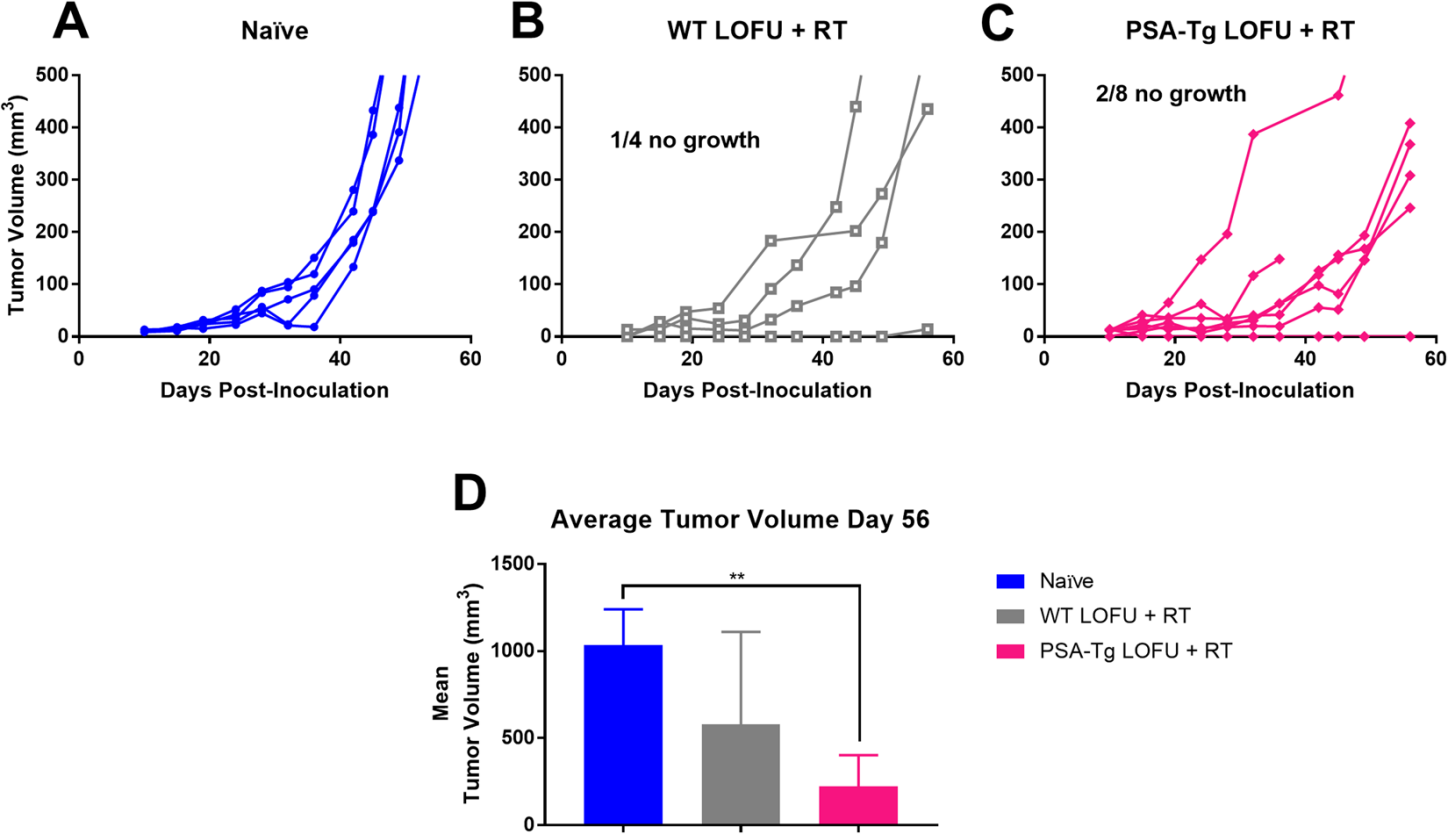
Combination Treatment with LOFU + RT protects mouse from tumor re-challenge. (**A**–**C**) Prior treatment with LOFU + RT inhibits secondary tumor growth. 5 naïve mice (**A**), 4 WT mice (**B**), and 8 PSA-Tg mice with primary tumors cured (**C**) were injected with TPSA23 cells and tumor volume was followed up to 60 days post inoculation. Naïve mice are wild-type C57BL/6 mice that have never been injected with TPSA23 prior to this experiment. (**D**) Combination LOFU + RT reduces average secondary tumor volume on day 56. Average re-challenge tumor volume with standard deviation (Kruskal-Wallis test p = 0.0056) One out of four WT mice and two out of eight PSA-transgenic mice showed complete tumor growth inhibition.

To determine if our treatment resulted in immunological memory, we re-challenged mice that were cured of their primary tumor after LOFU + RT therapy with TPSA23 cells on the contralateral flank and measured the tumor growth profile up to 60 days post-inoculation. Figure 6A–C depict the tumor growth in individual mice in each treatment group. At day 45 post-inoculation 6 out of 7 remaining PSA-transgenic mice showed significant growth delay [naïve: 307.6 mm^3^, WT: 184.8 mm^3^, PSA-Tg(6/7): 73.11 mm^3^, p = 0.01] and only 1 mouse showed complete lack of tumor memory [PSA-Tg(7/7: 128.6 mm^3^, p = 0.11]. By day 56 post-inoculation, 2 out of 8 PSA-transgenic mice had complete tumor growth inhibition (Fig. 6C). Re-challenge response in WT mice (Fig. 6B) was different, at day 43 post-inoculation, only 1 out of 4 showed tumor growth inhibition and persisted in its tumor inhibition response until the end of the experiment.

At day 56, the WT mice cured of primary tumor by LOFU + RT combination had a tumor growth delay of 52% and the PSA-transgenic mice cured of primary tumor by LOFU + RT combination had a tumor growth delay of 74% compared to naïve mice (Fig. 6D) indicative of immune memory generated by LOFU + RT treatment of the previously cured tumor. While both C57BL/6 (WT) and PSA-transgenic mice (C57BL/6 background) primary tumor-cured groups showed tumor growth retardation after rechallenge, only PSA-transgenic mice showed statistically significantly reduction in the tumor volume (p = 0.008, Dunn’s multiple comparisons) compared to the naïve group. While there was no statistically significant difference between the rechallenge tumor volume in wild-type rechallenged mice and naïve or PSA-transgenic mice, 1 out of 4 mice demonstrated complete immunological memory, while 3 out of 4 mice had a growth delay compared to non-treated. These experiments indicate that combination treatment of LOFU + RT not only resulted in primary tumor cure but also enhanced immunological memory, which resulted in significant inhibition of the secondary tumor growth.

## Discussion

This study highlights the significance of physical energy-based immune priming in combination with ablation for optimal induction of anti-tumoral immunity. Focal ablative therapies have been used primarily for local tumor ablation. However, they often fail to impact systemic disease. Our goal is to design focal therapies to modulate the tumor microenvironment to drive systemic anti-tumoral immunity. In this study, we demonstrate that LOFU-mediated immune priming stimulated a robust heat shock response with an increase in RNA, protein expression, and cell surface localization of HSPs in murine breast and prostate cancer cell lines. While LOFU alone was responsible for increased HSP mRNA and protein expression along with redistribution of HSPs to the cell surface, plasma HSP90α was only increased after LOFU + RT combination therapy. The addition of a non-ablative LOFU treatment enhanced the tumoricidal effects of RT with a significant increase in tumor growth inhibition and complete response in immune competent mice, due to activation of anti-tumoral immunity. The clinical significance of the prostate cancer-specific anti-tumoral immunity is highlighted by the induction of immunity and complete response in the PSA-transgenic mice, where PSA is a self-antigen. Surprisingly, LOFU enhanced the tumoricidal effects of radiation *in vitro* as evident by the decreased clonogenicity of the combination treatment when compared to the individual treatment. The mechanism of this enhancement is multi-faceted. One possible explanation is a LOFU-induced reduction in activated STAT3, which is known to mediate resistance to radiation-induced cell death. LOFU + RT induced PSA-specific activated CD62L-CD8+ T cells, reduced the levels of exhausted TIM3 + effector memory and activated T cells, and had increased tumor-specific CTL activity in splenocytes. Finally, mice that were cured after LOFU + RT exhibited immune memory and tumor growth inhibition upon re-challenge of TPSA23 cells.

The basis of LOFU-mediated immune priming lies with the heat shock response, which is an evolutionarily conserved cellular defense mechanism for promoting protein folding. Stress signals in the tumor microenvironment, including oxidative stress and heat can initiate a heat shock response with induced expression of molecular chaperones of the Hsp gene families. In both 4T1 and TPSA23, LOFU induced Hsp mRNA expression indicating that the acoustic stress pathway has a heat shock response component in tumor cells, possibly by inducing protein misfolding after LOFU treatment. An alternative stressor mechanism could be the direct effect of ultrasound on the plasma membrane. It is expected that the cell membrane would initially “sense” the ultrasound pulses. Since the thermal effects of a 1.5 second LOFU pulse is much lower than traditional hyperthermia treatment which lasts for 30–60 minutes, it is possible that LOFU could induce changes in the fluidity of membrane lipids and induce a heat shock-like response without significant protein denaturation. Mild hyperthermia has previously been shown to initiate a remodeling of lipids, redistribution of stress proteins, while maintaining membrane integrity^26^. A role for “membrane thermosensors” with a cell membrane-initiated heat shock response has been previously described in cancer cells^27^. A critical element for this process is the translocation of HSP proteins, such as, HSP70 and HSP90 on the cell surface of tumor cells^28^. Cell surface HSPs can be a target for natural killer cell-mediated cytolysis^29,30^ and can increase radiation sensitivity in tumor cells^31^. Thus, LOFU treatment can not only increase the tumoricidal effects of radiation but also can be viewed as an immune priming therapy that re-engineers the tumor microenvironment to drive systemic immunity.

Another characteristic of LOFU-mediated immune priming is the release of HSP-peptide complexes after tumor ablation. Cytosolic proteins are released into the extracellular compartment with eventual increase in plasma levels of HSPs after focal ablation of tumor. Tumor-infiltrating phagocytes, including DCs, can engulf these extracellular antigens and typically process and present antigenic peptides via MHC class II for CD4 + T helper cell activation through the endosomal pathway. For successful activation of CD8 + cytotoxic T lymphocytes (CTL), engulfed extracellular antigens need to transfer from the endosomes to the cytoplasm for proteasomal degradation for eventual cross-presentation to class I MHC via the endoplasmic reticulum antigen presentation pathway. Upon engulfment of extracellular antigens by DCs, HSP90 has been shown to promote the translocation of endosomal proteins into the cytosol, thus contributing to cross-presentation of extracellular antigens for CTL activation^32,33^. Therefore, it is possible that the increased release of HSP90-peptide complexes from LOFU + RT-treated tumor cells induced tumor-specific CD8 + CTL activation seen in our studies (Fig. 5C). While we have focused on extracellular HSP90 in our studies, tumor-derived HSP70-peptide complexes can also be cross-presented by human DCs^34^.

In summary, we describe a novel paradigm of immune priming ablation therapy using LOFU in combination with ablative RT for generating *in situ* tumor vaccine. With minimal side effects, non-invasive and non-ionizing LOFU treatment can be administered repeatedly with any ablative therapy, such as, RT, hormonal ablation, chemotherapy or immunotherapy during the course of prostate cancer therapy with the goal of inducing a systemic anti-tumoral immunity, thereby converting focal tumor ablation into systemic cure.

## Methods

### Cell lines

4T1 cells were cultured in DMEM high glucose (HyClone, GE Healthcare, South Logan, UT) with 10% of fetal bovine serum (FBS; Peak Serum, Wellington, CO) and 1% antibiotic/antimycotic (HyClone, GE Healthcare, South Logan, UT). A TPSA23 cell line was previously constructed from the murine prostate adenocarcinoma cell line to secrete human PSA, as described^16^. TPSA23 cells were cultured in DMEM high glucose, 5% FBS, 5% Nu-Serum IV (Corning, Corning, NY), 10 nM dehydroisoandrosterone (Sigma-Aldrich, St. Louis, MO), 5ug/ml bovine insulin (Sigma- Aldrich, St. Louis, MO), 1% antibiotic/antimycotic.

### *In vitro* treatment plan

Prior to *in vitro* treatment, cells were trypsinized with 0.05% trypsin, quantified with a hemocytometer and 2 × 10^6^ cells were distributed into 0.2 mL PCR tubes with 200ul of media. Cells were pelleted in the PCR tubes, submerged under degassed water over an ultrasound absorber. Treatment was performed on Philips’ Therapy and Imaging Probe System (TIPS, Philips Healthcare, Briarcliff, NY) in a grid fashion with a raster pattern to cover the entire pellet. All treatments were performed at 1 MHz frequency, 100 Hz pulse repetition frequency and a 1.5 second treatment time at each focal spot. Table 1 shows the other ultrasound parameters used. After treatment, cells were replated and incubated at 37 °C with 5% CO_2_ until the desired timepoint. All cells were routinely tested for mycoplasma contamination with the MycoAlert Kit (Lonza, Walkersville, MD).

**Table 1:** Focused Ultrasound Treatment Parameters.

Power (Watts)	Peak Negative Pressure (MPa)	Duty Factor (%)	Intensity (W/cm^2^)
1	2.11	50	97.8
3	3.05	50	204.3
5	3.75	50	308.9
**5**	**3.75**	**100**	**617.2**
7	4.34	50	413.7
9	4.81	50	508.2

Intensity calculated according to Wu and Nyborg^37^ with an attenuation coefficient (α) of 0.1 dB/cm, approximately that of soft tissue at 1 MHz, speed of sound (c) was 1584.5 m/s, approximately the speed of sound in soft tissue at 37 **°**C, density (ρ) approximated at 1070 kg/m^3^ for soft tissue, distance (x) was 0.3 cm, or half the focal length. Peak negative pressure values were obtained from the Philips’ TIPS software, based on initial calibration calculations. 99.9% duty factor is the greatest possible with the device and will be denoted as 100%. Bold indicates the treatment used *in vivo*.

### RNA isolation, qRT-PCR

For RNA isolation, at the desired time point, cells were harvested with 1 mL of TRIZol (Invitrogen, Carlsbad, CA) and stored at −20 °C until isolation. RNA isolation was performed according to manufacturer’s instructions (Invitrogen, Carlsbad, CA). cDNA was generated according to manufacturer’s instructions with the Verso cDNA synthesis kit (Thermo Fisher Scientific, Waltham, MA). qPCR was performed in 384-well plates with SYBR green as the marker on the Applied Biosystems 7900HT PCR System at the Genomics Core at Albert Einstein College of Medicine (Thermo Fisher Scientific, Waltham, MA). Primers (Table 2) were purchased from Eurofin Genomics. Data was analyzed using SDS version 2.4.

**Table 2:** Mouse HSP primers.

*Mouse Gene*	Sequence	Protein
*Hsph1 Forward*	CAGGTACAAACTGATGGTCAACA	HSP105/110
*Hsph1 Reverse*	TGAGGTAAGTTCAGGTGAAGGG	
*Hsp90aa1 Forward*	CCTAGGGTCGGAAGCCAT	HSP90α
*Hsp90aa1 Reverse*	GAGCAGGGCCGTAGGTTG	
*Hspa1a Forward*	GCACGTGGGCTTTATCTTCC	HSP72
*Hspa1a Reverse*	AACAAATCACATCAGCGGGG	
*Hspa1b Forward*	ACGTCTTGGCACTGTGTACT	HSP70-b
*Hspa1b Reverse*	AGGGTGGCAGTGTAGACATG	
*Hspb1 Forward*	TCACTGGCAAGCACGAAGAA	HSP27
*Hspb1 Reverse*	ATGGTGATCTCCGCTGACTG	
*Hsp70 Forward*	AGGGCATCGACTTCTACACA	HSP70
*Hsp70 Reverse*	ATCTGCGCCTTGTCCATCTT	
*Gapdh Forward*	GCAGTGGCAAAGTGGAGATT	GAPDH
*Gapdh Reverse*	GAATTTGCCGTGAGTGGAGT	

### Flow cytometry

For flow cytometry experiments, after LOFU treatment, cells were plated into a 96-well U-bottom plate and incubated for 4 hours. At the desired time, cells were washed with 0.5% BSA in PBS, blocked with CD16/CD32 (BD Biosciences, Billerica, MA) and stained with the antibody mix for 30 minutes at 4 °C. The following antibodies were used in the *in vitro* mix: HSP70-FITC (1:200), HSP60-AlexaFluor405 (1:100), HSP90-AlexaFluor700 (1:100) [all from Novus Biologicals, Littleton, CO] and Live/Dead Fixable Blue Dead Stain kit (Invitrogen, Carlsbad, CA). After staining, cells were washed and resuspended for acquisition on the BD LSRII at the Flow Cytometry Core at Albert Einstein College of Medicine and analyzed with FlowJo Software v10.

### HSP70 and HSP90α ELISA

Four, eight and twenty-four hours after LOFU or sham treatment cell lysates were generated using RIPA buffer (MilliporeSigma, Burlington, MA) with protease/phosphatase inhibitors and stored at −80 °C until ready for use. The cell culture supernatant was also stored at −80 °C for future use. Total protein concentration was measured using spectrophotometry (SpectraDrop, SpectraMax M3, Molecular Devices, Sunnyvale, CA). HSP70/HSPA1A ELISA was performed following manufacturer’s instructions using 50 µL of lysate or cell culture supernatant.

Plasma from treated mice was isolated the day after the end of treatment and frozen at −80 °C until ready for HSP90α ELISA. Manufacturer’s instructions (MyBioSource, San Diego, CA) were followed using 50 µL of plasma.

### Clonogenic assay

After LOFU treatment or sham treatment, 500 cells were plated in 6-well plates and incubated for 2 hours. The indicated plates were irradiated at 2 Gy using a Shepard Mark I Cesium 137 irradiator and incubated for 7 days. Media was removed from the plates which were then stained with 0.1% crystal violet in 10% phosphate buffered formalin for 2 minutes before rinsing with water. Once the plates were dried, colonies with greater than 50 cells were counted, and the survival fraction was calculated using the plating efficiency from non-treated cells.

### *In Vivo* model & treatment

All mouse protocols were performed in accordance with policies previously approved by the Institutional Animal Care and Use Committee of Albert Einstein College of Medicine. Wild-type C57BL/6 male mice and athymic nude (BALB/c background) male mice were purchased from Charles River Laboratories (Wilmington, MA). A breeding pair of PSA-transgenic mice (PSA-Tg), which exhibits prostate-specific expression of the entire human PSA gene, was gift from Dr. Frelinger^24^, and the colony was maintained at the Albert Einstein College of Medicine.

For tumor inoculation, TPSA23 cells were harvested, counted and resuspended in PBS at 20 × 10^6^ cells/mL. Fifty microliters of the cell suspension was injected into the right flank of shaved male C57BL/6 mice, anesthetized under continuous inhaled isoflurane. When the tumors reach 4–6 mm in diameter, treatment began. The area around the tumor was epilated with Nair (Church & Dwight, Ewing Township, NJ) and tumor dimensions measured with calipers prior to ultrasound treatment. Mice were anesthetized with isoflurane for treatments. Tumors were pulled away from the mouse’s body and acoustically coupled to a gel pad (Aquasonic, Parker Laboratories, Fairfield, NJ) on top of an ultrasound absorber. Ultrasound treatments were performed with the same ultrasound device as *in vitro* treatments at 1 MHz frequency, 100% duty cycle, 5W and 1.5 second treatment time per focal spot in a grid pattern with a maximal temperature of 45 °C achieved. Radiation treatment (10 Gy) was performed on the Small Animal Radiation Research Platform (XStrahl Medical, Suwanee, GA) 2–3 hours following ultrasound treatment due to our prior studies using this treatment scheme^13^ and other studies demonstrating reduction in phosphorylated-STAT3 after focused ultrasound treatment^17,18^. Treatments were repeated after about 48 hours to allow time for normal tissue recovery. Tumors were measured 1–2 times per week and tumor volume was calculated with the following formula $V=length\times width\times height\times \frac{3.1456}{6}$^35^. If used for tumor growth and survival studies, mice were euthanized when tumor volume reached 1000 mm^3^ in accordance with IACUC protocols.

### Tissue digestion

Tissues were harvested after mice were humanly euthanized. Spleens were digested by mechanical force, filtered through a 40 µm strainer and RBC were lysed with ACK lysing buffer. Tumors were chopped into small pieces, added to 100 U/mL each of collagenase I & IV (Gibco, Thermo Fisher Scientific, Waltham, MA) and Dnase I (Roche, Basel, Switzerland), heated at 37 °C, with mechanical digestion. The cell suspension was filtered through 70 µm strainer prior to staining.

### Tumor rechallenge

At least 30 days after tumor cure, 5 ×10^5^ TPSA23 cells were injected subcutaneously on the left flank. Tumor measurements were taken as described above.

### Tumor growth delay calculation

Tumor growth delay was calculated from the rechallenge tumors as the ratio of the difference in median tumor volume (V_T_) between the control and treated group and the median of the control group tumor volume $({{\rm{V}}}_{{\rm{c}}}) \% TGD=\frac{{V}_{C}-{V}_{T}}{{V}_{C}}\cdot 100$^36^.

### Immune Studies

Seven days after the end of treatment, at the end of peak T cell activation range (typically 5–7 days), splenocytes were isolated from treated mice and used for several assays. Flow cytometry for PSA-specific (MHC Class I restricted pentamer, ProImmune, Oxford, UK) and exhausted T cells (TIM3+) was performed. An LDH cytotoxicity assay was performed after overnight incubation of 20,000 mitomycin C treated tumor cells with the same number of splenocyte according to manufacturer’s instructions (Pierce LDH Cytotoxicity, Pierce Biotechnology, Waltham, MA).

### Statistical analysis

All *in vitro* experiments were performed in biological triplicates and three independent experiments. For *in vivo* tumor growth experiments, at least 6 mice were used per experimental group and for immunological endpoints, at least 4 mice per group was used. All experiments were performed on three independent occasions. All graphs display standard deviation. For comparisons of 3 or more groups, a non-parametric ANOVA (Kruskal-Wallis) test with multiple comparisons comparing the mean of experimental groups to the mean of the control groups (Dunn’s test) was performed using GraphPad Prism For experiments with only two groups, a Mann-Whitney test was used for statistical analysis and for Fig. 4, log-rank analysis was performed. The Fisher’s exact test for primary tumor cure was calculated online. Statistically significant p values are noted as: *≤0.05, **≤0.01, ***≤0.001.
